# Impact of glucagon-like peptide 1 analogs on cognitive function among patients with type 2 diabetes mellitus: A systematic review and meta−analysis

**DOI:** 10.3389/fendo.2022.1047883

**Published:** 2022-10-28

**Authors:** Sisi Luan, Wenke Cheng, Chenglong Wang, Jianhong Gong, Jianbo Zhou

**Affiliations:** ^1^ Department of Endocrinology, Beijing Tongren Hospital, Capital Medical University, Beijing, China; ^2^ Medical Faculty, University of Leipzig, Leipzig, Germany; ^3^ Plastic Surgery Hospital, Peking Union Medical College, Chinese Academy of Medical Sciences, Beijing, China; ^4^ Beijing Tongren Hospital, Capital Medical University, Beijing, China

**Keywords:** glucagon-like peptide 1, cognitive function, T2DM, cardio-cerebrovascular disease, meta-analysis

## Abstract

**Background:**

Diabetes is an independent risk factor for cognitive impairment. However, little is known about the neuroprotective effects of glucagon-like peptide 1 (GLP-1) analogs on type 2 diabetes mellitus (T2DM). Herein, we assessed the impact of GLP-1 analogs on the general cognitive functioning among patients with T2DM.

**Methods:**

Relevant studies were retrieved from PubMed, Embase, Cochrane Central Register of Controlled Trials (CENTRAL), and ClinicalTrials.gov databases from their inception till June 30, 2022, without any language restrictions. For continuous variables, the mean and standard deviation (SD) were extracted. Considering the heterogeneity in general cognitive functioning assessments among the pooled studies, the standardized mean differences (SMDs) with corresponding 95% confidence intervals (CIs), were calculated.

**Results:**

Five studies including 7,732 individuals with T2DM were selected for the meta-analysis. The use of GLP-1 analogs exerted no significant effects on the general cognitive functioning in self-controlled studies (SMD 0.33, 95% CI -0.03 to 0.69). Subgroup analyses among the self-controlled studies based on age and history of cardio-cerebrovascular disease showed that GLP-1 analogs significantly improved the general cognitive functioning in T2DM patients younger than 65 years (SMD 0.69, 95% CI 0.31 to 1.08) or those without cardio-cerebrovascular diseases (SMD 0.69, 95% CI 0.31 to 1.08). Similarly, differences in the general cognitive functioning for GLP-1 analogs between treated and non-treated patients with T2DM were significant in subgroups with patients younger than 65 years (SMD 1.04, 95% CI 0.61 to 1.47) or those with no history of cardio-cerebrovascular diseases (SMD 1.04, 95% CI 0.61 to 1.47).

**Conclusion:**

Limited evidence suggests that the use of GLP-1 analogs exerts no significant effects on general cognitive functioning but may be beneficial for patients with T2DM younger than 65 years or those without a history of cardio-cerebrovascular diseases. Further prospective clinical studies with large sample sizes are needed to validate these findings.

**Systematic Review Registration:**

www.inplasy.com, identifier 202260015.

## Introduction

Cognitive impairment defined as difficulty in processing thoughts, can lead to memory loss, decision-making difficulties, inability to concentrate and learning difficulties, and has emerged as one of the major public health challenges of our time (1). Type 2 diabetes mellitus (T2DM) is associated with reduced performance in multiple domains of cognitive functioning (2, 3). Although the exact pathophysiology of cognitive impairment in T2DM remains unclear, hypoglycemia, insulin resistance, related end products, complications associated with diabetes, and physical/psychological status may play significant roles (4, 5). With the emergence of new antidiabetic agents exerting multiple therapeutic effects, understanding their potential impact on cognitive functioning along with hypoglycemic activities may be valuable.

Glucagon-like peptide-1 (GLP-1), a peptide hormone from the intestinal tract, plays a central role in the maintenance of postprandial glucose homeostasis through coordinating insulin secretion, food intake, and gut motility (6, 7). Native GLP-1 is degraded within 2-3 min in the circulation; various GLP-1 analogs have been developed to exert prolonged *in vivo* activity for the treatment of T2DM and obesity (8). In addition to improved glycemic control, the protective effects of GLP-1 analogs on cognitive impairment in diabetes have been supported by compelling experimental evidence. The possible molecular mechanisms by which GLP-1 analogs improve cognitive functioning include reduced oxidative damages (9), declining inflammatory responses (10), reduced apoptotic death of neuronal structures (11), facilitating insulin signaling (12), crossing the blood-brain barrier, and directly modulating the central area involved in learning and memory (13). However, the results of clinical studies on the neuroprotective effects of GLP-1 analogs on T2DM patients remain controversial. Herein, we aimed to evaluate the association of GLP-1 analogs and general cognitive functioning among patients with T2DM.

## Material and methods

### Study design

The protocol and report of this study followed the “Preferred Reporting Items for Systematic Reviews and Meta-Analyses” (PRISMA) statement (14) and were registered in the INPLASY-International Platform of Registered Systematic Review and Meta-analysis Protocols (identifier: INPLASY 202260015). The PubMed, Embase, Cochrane Central Register of Controlled Trials (CENTRAL), and ClinicalTrials.gov databases were queried for relevant studies published up to June 30, 2022, without any language restrictions. A detailed search strategy is provided in the **Supplement**, and the reference lists of eligible articles were queried further for pertinence.

### Inclusion and exclusion criteria

Inclusion and exclusion criteria were set according to PICOS (Population, Intervention, Comparison, Outcomes, and Study Design).

Inclusion criteria were as follows: (1) the population comprised adult subjects diagnosed with T2DM at the baseline; (2) GLP-1 analogs of single formulation rather than a combination at fixed doses; (3) GLP-1 analogs compared with no use of GLP-1 analogs, placebo, or self-control before treatment; (4) the duration of GLP-1 analogs use was 12 weeks or more; (5) the quantitative measures of general cognitive functioning were assessed by Mini-mental State Examination (MMSE) or Montreal Cognitive Assessment (MoCA), and (6) the design was limited to prospective studies.

Exclusion criteria were as follows: (1) the publication was a review, case report, basic research, or letter to the editor; (2) studies with missing MMSE or MoCA scores; (3) the authors could not provide valid data upon request, and (4) duplicated data.

### Data extraction and quality assessment

Two investigators (SS-L and CL-W) independently extracted the data from the five included studies using the same standardized method, including the quality, population characteristics, year of publication, and outcomes. Two reviewers (SS-L and WK-C) independently assessed the risk of bias in the included cohort studies by awarding stars in each domain following the guidelines of the Newcastle-Ottawa Quality Assessment Scale criteria (NOS) (15). The risk of bias in included random controlled trials was assessed independently by the above reviewers using the revised Cochrane risk-of-bias tool for randomized clinical trials (16). Any disagreements among the investigators were discussed with the other authors to arrive at a consensus.

### Statistical analysis

The sample number, mean MMSE or MoCA values, and SD from the cognitive assessment scale were extracted from the selected studies to assess the differences in general cognitive functioning for GLP-1 analogs between the treatment and control groups. Considering the heterogeneities in the experimental measurement methods, the standardized mean difference (SMD) was utilized. In studies reporting median values and interquartile ranges with a large sample size (> 100/group), the median values were treated as means, and SD was calculated as follows: 75^th^ minus 25^th^ percentiles divided by 1.35 (17). GetData Graph Digitizer 2.26 was used to obtain data from figures in case the original data were not available upon request. All statistical analyses were performed on Stata 12.0E.

## Results

### Literature search outcomes and validity assessment

**Figure 1:**
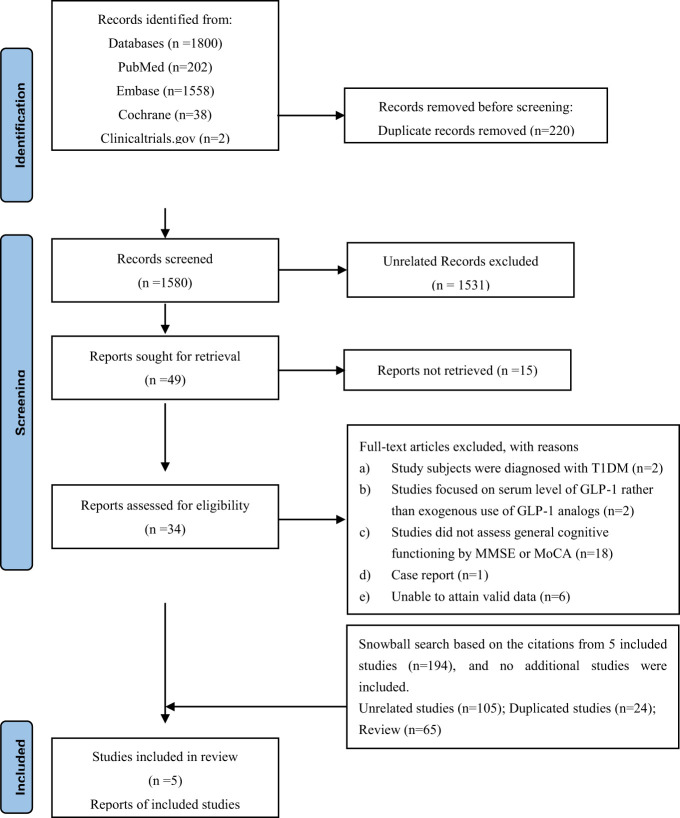
Flow chart of study selection for the meta-analysis.

We identified 1800 potentially relevant reports, of which 220 were excluded as these were duplicates. We screened the titles and abstracts of the remaining 1580 manuscripts. Subsequently, 1531 publications were removed, as these were reviews, letters or conference abstracts, or basic research. A total of 15 articles were excluded as these lacked relevant information or the full publication was absent. Finally, 34 articles were eligible for full-text review and data assessment. A total of 5 studies comprising three randomized controlled trials (18–20) and two prospective cohort studies (21, 22) that met the inclusion criteria were analyzed (**Figure 1**). Consequently, a “snowball search” was performed based on the citation lists of all the included studies. In the 5 initially identified studies, 194 citations were extracted, whereby 105 were irrelevant for the meta-analysis, 24 were duplicates, and 65 were reviews. Ultimately, no additional studies were included in the analysis. The baseline characteristics of the eligible studies and participants are shown in **Table 1** and **Table S1**.

**Table 1:** Characteristics of the five prospective studies.

Author, year	Sample number	GLP-1 RA category	Control	Age	History of cardio-cerebrovascular disease	Treatment duration	General cognitive assessments
Cheng et al., 19	36	Liraglutide	Self-control, non-GLP-1 analog treated	GLP-1 analog group: 51.9 ± 10.2	No	16 weeks	MMSE, MoCA
Li et al., 21	47	Liraglutide	Self-control, non-GLP-1 analog treated	GLP-1 analog group: 55.0 ± 11.9	No	12 weeks	MMSE
Cukierman-Yaffe et al., 18	7570	Dulaglutide	Self-control, non-GLP-1 analog treated	GLP-1 analog group: 65.5 ± 6.4	Yes	60 months	MoCA
Wang et al., 20	60	Liraglutide	Self-control, non-GLP-1 analog treated	GLP-1 analog group: 66.1 ± 5.9	Yes	6 months	MMSE, MoCA
Zhang et al., 22	19	Liraglutide, Exenatide	Self-control	52.1 ± 10.2	No	3 months	MoCA

### Quality assessment

Quality assessment results of cohort studies were at a scale of 8 to 9 using the NOS evaluation tool. The quality of the included studies was high as shown in **Table S2**. All random controlled trials had a low or unclear risk of bias across the 5 evaluated domains based on the revised Cochrane risk-of-bias tool (**Figure S1**, **Figure S2** **)**.

### Association of GLP-1 analogs with general cognitive functioning

**Figure 2:**
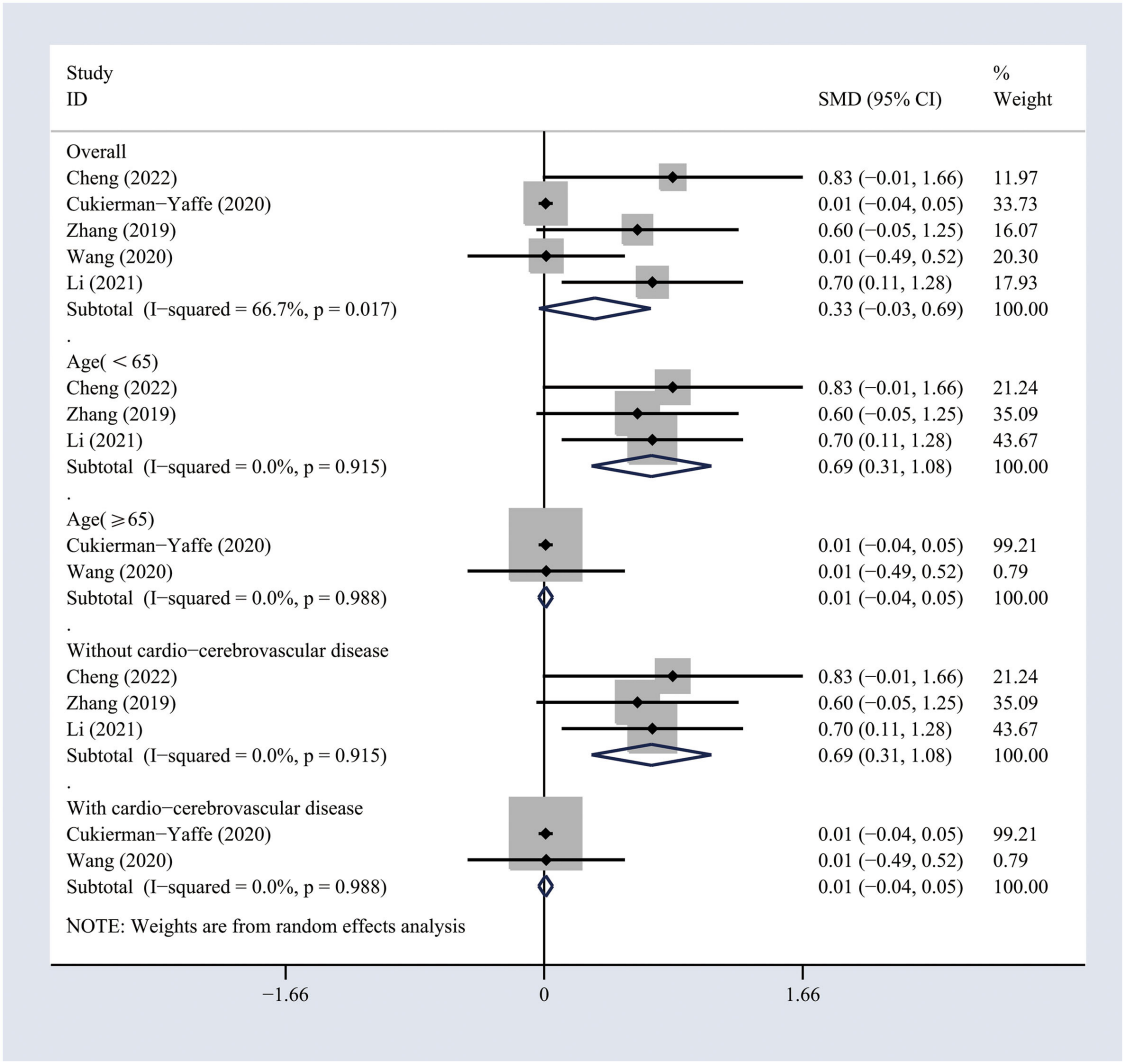
Forest plots of random-effects meta-analysis (between-study variance estimator: DerSimonian and Laird (DL)) for the differences in cognitive function before and after treatment with GLP-1 analogs. Shown are the standardized mean differences (SMDs) together with their 95% confidence intervals (CIs) as effect measures.

**Figure 3:**
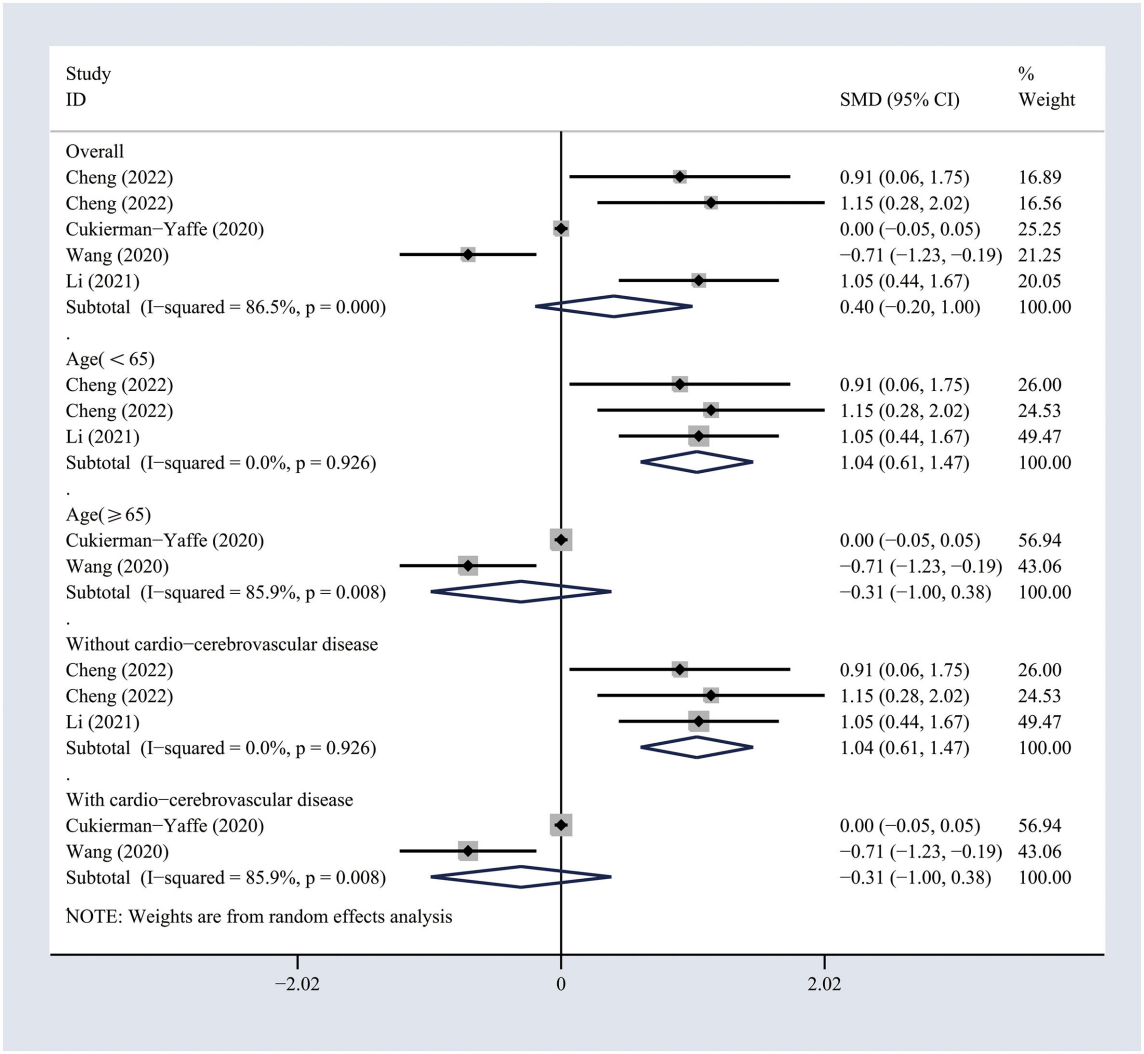
Forest plots of random-effects meta-analysis (between-study variance estimator: DerSimonian and Laird (DL)) for the differences in cognitive function between treated and non-treated groups with GLP-1 analogs. Shown are the SMDs together with their 95% CIs as effect measures.

The included studies comprising 7732 individuals focused on the impact of treatment with GLP-1 analogs on the cognitive functioning of patients with T2DM (18–22). The use of GLP-1 analogs showed no significant effects on the general cognitive functioning across the self-controlled studies (SMD 0.33, 95% CI -0.03 to 0.69; I^2 =^ 66.7%) (**Figure 2** **)**. Sensitivity analyses were performed by excluding one study at a time, and the pooled results of the cognitive assessment changed slightly when the study conducted by Cukierman et al. was excluded (**Figure S3**). Similarly, no significant effects of GLP-1 analogs on the general cognitive functioning as compared to the non-GLP-1 analog-treated group (SMD 0.4, 95% CI -0.2 to 1; I^2 =^ 86.5%) (**Figure 3** **)** were observed. Sensitivity analyses were conducted by excluding one study at a time, and the pooled results of general cognitive functioning were stable (**Figure S4**). The pooled results obtained from the random model were the same.

### Subgroup analyses

To investigate the potential sources of heterogeneity, subgroup analyses were conducted based on the age and history of cardio-cerebrovascular diseases. The results suggested that GLP-1 analogs significantly improved the general cognitive functioning in T2DM patients younger than 65 years (SMD 0.69, 95% CI 0.31 to 1.08; I^2 =^ 0%) or in those without cardio-cerebrovascular diseases (SMD 0.69, 95% CI 0.31 to 1.08; I^2 =^ 0%) across self-controlled studies (**Figure 2** **)**. Similarly, the differences in general cognitive functioning between GLP-1 analog-treated and non-treated patients with T2DM were significant in the subgroups of patients younger than 65 years (SMD 1.04, 95% CI 0.61 to 1.47; I^2 =^ 0%) or those with no history of cardio-cerebrovascular diseases (SMD 1.04, 95% CI 0.61 to 1.47; I^2^ = 0%) (**Figure 3** **)**. The results of subgroup analyses indicated that the impact of GLP-1 analogs on general cognitive functioning in patients with T2DM may be related to the age of the patient and the history of cardio-cerebrovascular diseases.

## Discussion

Overall, our analysis comprising 7,732 individuals showed that the use of GLP-1 analogs had no significant effect on general cognitive functioning but may serve as a protective factor in patients with T2DM younger than 65 years or those with no history of cardio-cerebrovascular diseases, thereby, suggesting dependency on the extent of neuropathy.

Despite the compelling experimental evidence for the neuroprotective effects of GLP-1 analogs (23–27), their therapeutic effects on the general cognitive function of T2DM patients remain largely unclear. As most experiments were conducted *in vitro* or in rodents, the neuroprotective effects of GLP-1 analogs may not be ideal in humans. Another possibility is that the MMSE and MoCA scores are not sensitive enough to detect the improvement in certain aspects of cognitive functions in T2DM patients following treatment with GLP-1 analogs, and additional and extensive tests are needed (28, 29). Furthermore, cognitive impairment is long-term, whereby some patients may progress to the stage of dementia, while others remain stable or show complete recovery of function. Therefore, a follow-up study spanning several months may not be able to capture enough information on the long-term effects of the use of GLP-1 analogs.

Our analysis indicated that GLP-1 analogs may exert a protective impact on cognitive impairment specifically among younger patients, thereby highlighting the importance of stratifying the patients with cognitive impairment by severity and the duration of the disease when studying the therapeutic potential of medications in clinical settings. For older patients with more severe diseases spanning decades, reversing it is difficult and, may need a longer time for follow-up. Aging has a profound influence over various biological processes, including aberrant autophagy, mitochondrial dysfunction, cellular senescence, epigenetic changes, cerebrovascular dysfunction, inflammation, and lipid dysregulation, that play critical roles in neurodegenerative diseases (30). Therefore, aging may affect some targets of the GLP-1 analogs, thereby affecting the neural protective function and making them less effective among aged T2DM patients.

Likewise, our results suggested that GLP-1 analogs may be more effective against cognitive impairment among T2DM patients without cardio-cerebrovascular diseases. Vascular cognitive impairment refers to the contribution of vascular pathology to any severity of cognitive impairment (31). Although diabetes is a major risk factor for vascular diseases, vascular pathology may cause cognitive impairment with slightly different mechanistic settings, that are less sensitive to GLP-1 analogs as compared to cognitive impairment associated with insulin resistance, glucose fluctuations, or other mechanisms not involving cerebral vessels. Although previous studies have confirmed the cardio-cerebrovascular actions and therapeutic potential (32–34), existing clinical findings suggest that GLP-1 analogs are ineffective against cognitive decline in the presence of cardio-cerebrovascular disease. Thus, confirming the efficacy of GLP-1 analogs for the improvement of cognitive functioning in T2DM patients older than 65 years and those with a history of cardio-cerebrovascular disease is warranted.

Although our meta-analysis provided valuable evidence on the relationship between GLP-1 analogs and cognitive functions, the strengths of our study and its limitations warrant mention. To the best of our knowledge, this is the first meta-analysis to verify the effects of GLP-1 analogs on cognitive functions in patients with T2DM. Unexpectedly, we found the impact of GLP-1 analogs on the general cognitive functioning in T2DM patients may be associated with the age and history of cardio-cerebrovascular diseases. These results provide new perspectives for the treatment of cognitive impairment in T2DM patients in clinical settings. Furthermore, we explicitly defined our inclusion criteria, developed a comprehensive search strategy, performed a duplicate quality assessment of enrolled studies, extracted available data, and transformed these uniformly.

Nonetheless, certain limitations should be considered. 1) Due to relatively insufficient data, only five prospective studies including 7732 individuals with T2DM were selected, and thus, further research with larger sample sizes is required. 2) The heterogeneity of results may be caused by the differences in characteristics of subjects, duration of disease, complications, and form or dose of GLP-1 analogs. Age and cardio-cerebrovascular complications were identified as possible sources of heterogeneity through subgroup analyses. However, owing to the insufficient number of existing studies, further subgroup analyses were not conducted to detect other potential sources of heterogeneity. 3) This review was predominated by the study performed by Cukierman et al. (7570/7732, 97.90%) among the five included studies. The robustness of the review was determined by the quality and research integrity of this study. 4) Due to insufficient clinical research data, the meta-analysis did not include studies conducted with semaglutide, a GLP-1 analog, directly accessing the brainstem, septal nucleus, and hypothalamus. It can interact with the brain through the circumventricular organs and several selected sites adjacent to the ventricles, thereby exerting a prominent impact on the central nervous system (35). 5) Finally, in clinical practice, diabetic patients usually use more than one type of antidiabetic agent. The impact of combined antidiabetic therapy on cognitive function remains unclear.

## Conclusion

Based on the existing limited evidence, our findings indicated that the use of GLP-1 analogs had no significant effects on the general cognitive functioning among patients with T2DM but may be beneficial for those younger than 65 years or without a history of cardio-cerebrovascular diseases, and thus, might be dependent on the extent of neuropathy. Nevertheless, multicenter, multi-regional, and large-sample studies are needed to complement and further validate these findings in the future.

## Data availability statement

The original contributions presented in the study are included in the article/**Supplementary Material**. Further inquiries can be directed to the corresponding author.

## Author contributions

SL and JZ contributed to the conception and study design. SL, WC, and CW performed data acquisition, statistical analysis, and interpretation. SL drafted the manuscript. SL, WC, JG, and JZ critically revised the manuscript. All authors gave their final approval.

## Funding

This work was supported by grants from the National Natural Science Foundation of China (grant numbers: 82100828, 82270866), and Excellent Talents in Economic-Technological Development District of Beijing.

## Acknowledgments

The authors are solely responsible for the design and conduct of this study, analyses, drafting and editing of the manuscript, and its final contents.

## Conflict of interest

The authors declare that the research was conducted in the absence of any commercial or financial relationships that could be construed as a potential conflict of interest.

## Publisher’s note

All claims expressed in this article are solely those of the authors and do not necessarily represent those of their affiliated organizations, or those of the publisher, the editors and the reviewers. Any product that may be evaluated in this article, or claim that may be made by its manufacturer, is not guaranteed or endorsed by the publisher.
