# Supplementary material for: Impact of glucagon-like peptide 1 analogs on cognitive function among patients with type 2 diabetes mellitus: A systematic review and meta−analysis
Source: Front Endocrinol (Lausanne). 2022 Oct 28;13:1047883. doi: 10.3389/fendo.2022.1047883 (PMC9650490; doi:10.3389/fendo.2022.1047883)
Supplement: Supplementary file 1 [file DataSheet_1.docx]

**Search strategy**

PubMed:

((((((type 2 diabetes) OR (Type 2 Diabetes Mellitus)) OR (Diabetes Mellitus, Type II)) OR (Diabetes, Type 2)) OR (Diabetes Mellitus, Type 2[MeSH Terms])) AND (((((((Dulaglutide) OR (Albiglutide)) OR (Semaglutide)) OR (GLP-1)) OR (Exenatide[MeSH Terms])) OR (Liraglutide[MeSH Terms])) OR (Glucagon-Like Peptide 1[MeSH Terms]))) AND ((((Dementia[MeSH Terms]) OR (Cognitive Dysfunction[MeSH Terms])) OR (Cognition[MeSH Terms])) OR ((((cognitive impairment) OR (cognitive decline)) OR (cognitive function)) OR (Mild Cognitive Impairment)))

Embase：

('non insulin dependent diabetes mellitus'/exp OR 'type 2 diabetes' OR 'Type 2 Diabetes Mellitus' OR 'Diabetes Mellitus, Type II' OR 'Diabetes, Type 2' OR 'Diabetes Mellitus, Type 2') AND ('glucagon like peptide 1'/exp OR 'glucagon like peptide 1' OR 'exendin 4'/exp OR 'exendin 4' OR 'liraglutide'/exp OR 'liraglutide' OR 'semaglutide'/exp OR 'semaglutide' OR 'albiglutide'/exp OR 'albiglutide' OR 'dulaglutide'/exp OR 'dulaglutide' OR 'glp-1' OR 'exenatide') AND ('dementia'/exp OR 'cognitive defect'/exp OR 'cognitive dysfunction' OR 'cognition'/exp OR 'cognitive impairment' OR 'cognitive decline'/exp OR 'cognitive function' OR 'mild cognitive impairment'/exp)

Cochrane Library (CENTRAL):

#1 ("type 2 diabetes mellitus"):ti,ab,kw

#2 (Diabetes Mellitus, Type II):ti,ab,kw

#3 (Diabetes, Type 2):ti,ab,kw

#4 (type 2 diabetes):ti,ab,kw

#5 MeSH descriptor: [Diabetes Mellitus, Type 2] explode all trees

#6 #1 OR #2 OR #3 OR #4 OR #5

#7 MeSH descriptor: [Exenatide] explode all trees

#8 MeSH descriptor: [Liraglutide] explode all trees

#9 MeSH descriptor: [Glucagon-Like Peptide 1] explode all trees

#10 (Dulaglutide):ti,ab,kw

#11 (Albiglutide):ti,ab,kw

#12 (Semaglutide):ti,ab,kw

#13 (GLP-1):ti,ab,kw

#14 #7 OR #8 OR #9 OR #10 OR #11 OR #12 OR #13

#15 MeSH descriptor: [Dementia] explode all trees

#16 MeSH descriptor: [Cognitive Dysfunction] explode all trees

#17 MeSH descriptor: [Cognition] explode all trees

#18 (cognitive impairment):ti,ab,kw

#19 (cognitive decline):ti,ab,kw

#20 (cognitive function):ti,ab,kw

#21 (Mild Cognitive Impairment):ti,ab,kw

#22 #15 OR #16 OR #17 OR #18 OR #19 OR #20 OR #21

#23 #6 AND #14 AND #22

Clinicaltrials.gov:

#1 Condition or disease: Diabetes Mellitus, Type 2

#2 Other terms: ((Dementia[MeSH Terms]) OR (Cognitive Dysfunction[MeSH Terms])) OR (Cognition[MeSH Terms])) OR ((((cognitive impairment) OR (cognitive decline)) OR (cognitive function)) OR (Mild Cognitive Impairment)))

#3 Study types: interventional

#4 Study results: studies with results

**STable 1. Characteristics of the included studies.**

| Study | Year | Sample | GLP-1 RA | Control | Treatment duration | Age | | History of cardio-cerebrovascular disease | MMSE | | MoCA | |
| --- | --- | --- | --- | --- | --- | --- | --- | --- | --- | --- | --- | --- |
|  |  |  |  |  |  | GLP-1 RA | Control |  | GLP-1 RA | Control | GLP-1 RA | Control |
| Cheng et al. | 2022 | 24 | Liraglutide | Dapagliflozin | 16 weeks | 51.9±10.2 | 57.0±9.5 | No | 29.14±0.45 | 29.14±0.45 | 27.55±0.57 | 26.98±0.68 |
| Cheng et al. | 2022 | 24 | Liraglutide | Acarbose | 16 weeks | 51.9±10.2 | 56.4±8.9 | No | 29.14±0.45 | 28.91±0.57 | 27.55±0.57 | 26.76±0.79 |
| Cheng et al. | 2022 | 12 | Liraglutide | Self-control | 16 weeks | 51.9±10.2 | | No | 28.68±0.45 | 29.14±0.45 | 26.98±0.79 | 27.55±0.57 |
| Li et al. | 2021 | 47 | Liraglutide | Non-GLP-1 analog | 12 weeks | 55.0±11.9 | 59.5±7.4 | No | 28.96±1.00 | 27.48±1.73 | — | — |
| Li et al. | 2021 | 47 | Liraglutide | Self-control | 12 weeks | 55.0±11.9 | | No | 27.92±1.86 | 28.96±1.00 | — | — |
| Cukierman-  Yaffe et al. | 2020 | 6348 | Dulaglutide | Placebo | 60 months | 65.5±6.4 | 65.5±6.4 | Yes | — | — | 24·62±4.44 | 24·62±4.53 |
| Cukierman-  Yaffe et al. | 2020 | 4448 | Dulaglutide | Self-control | 60 months | 65.5±6.4 | | Yes | — | — | 24.58±4.09 | 24·62±4.44 |
| Wang et al. | 2020 | 60 | Liraglutide | Sitagliptin | 6 months | 66.1±5.9 | 67.2±7.1 | Yes | 22.70±1.80 | 26.83±0.91 | 22.23±2.18 | 23.73±2.03 |
| Wang et al. | 2020 | 30 | Liraglutide | Self-control | 6 months | 66.1±5.9 | | Yes | 25.37±1.16 | 22.70±1.80 | 22.20±2.35 | 22.23±2.18 |
| Zhang et al. | 2019 | 19 | Liraglutide,  Exenatide | Self-control | 3 months | 52.1±10.2 | | No | — | — | 26.60±2.40 | 27.90±1.90 |

**STable2. Risk of bias assessment (Newcastle-Ottawa Quality Assessment Scale criteria).**

| Study | Selection | Comparability | Outcome | Quality score |
| --- | --- | --- | --- | --- |
| Li, 2021 | **** | * | *** | 8 |
| Zhang, 2019 | **** | ** | *** | 9 |


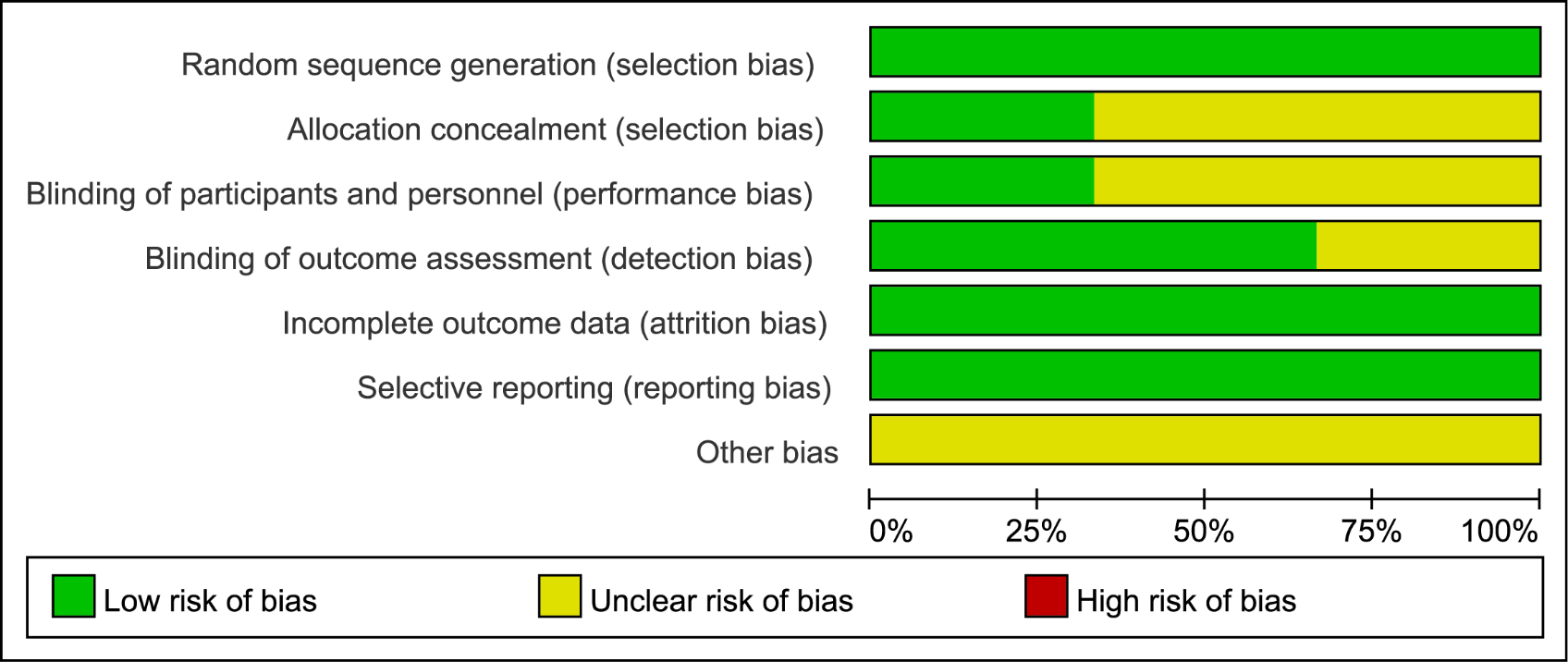


**SFigure1. Risk of bias graph: review authors' judgements about each risk of bias item presented as percentages across all included studies.**


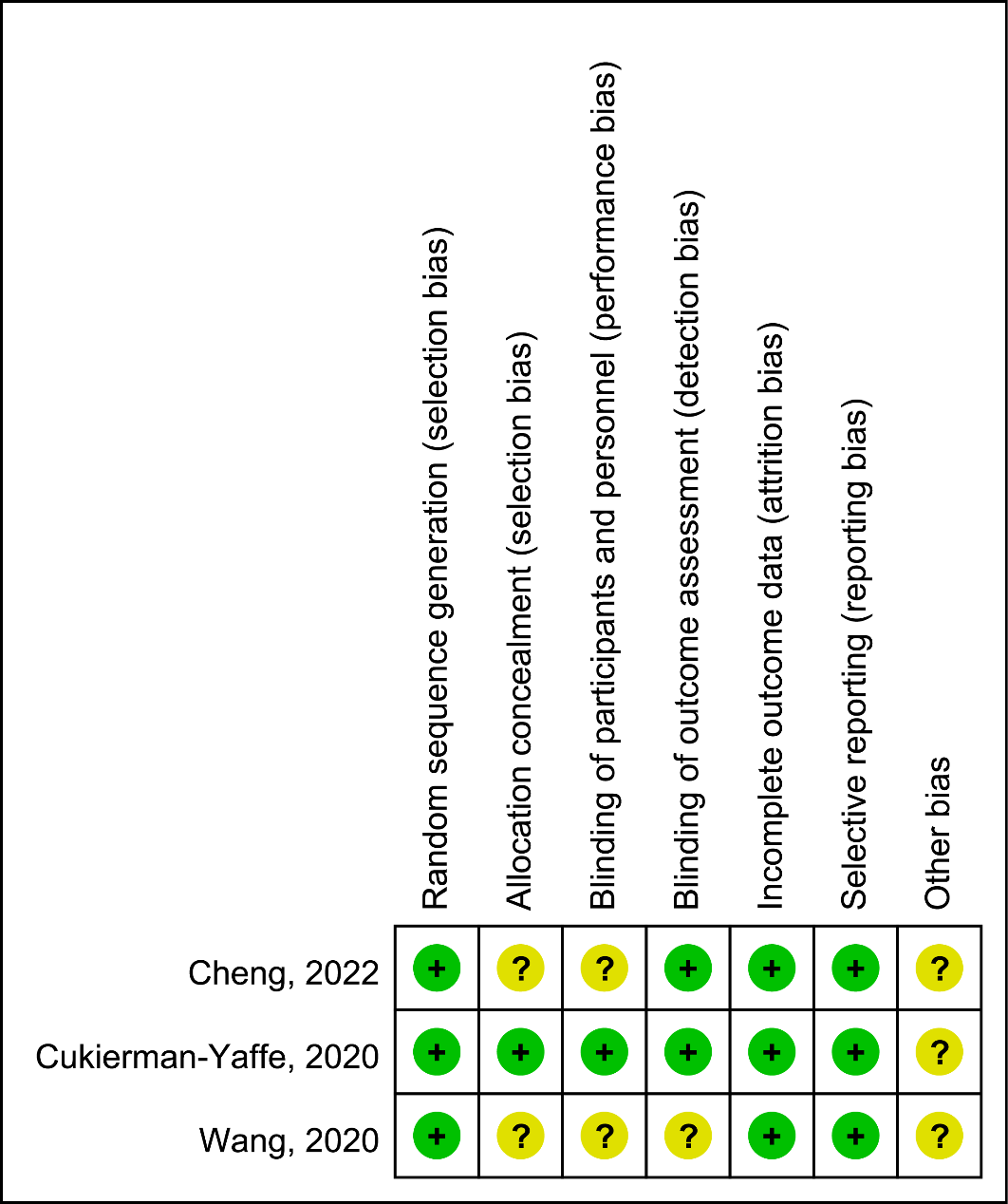


**SFigure2. Risk of bias summary: review authors' judgements about each risk of bias item for each included study.**


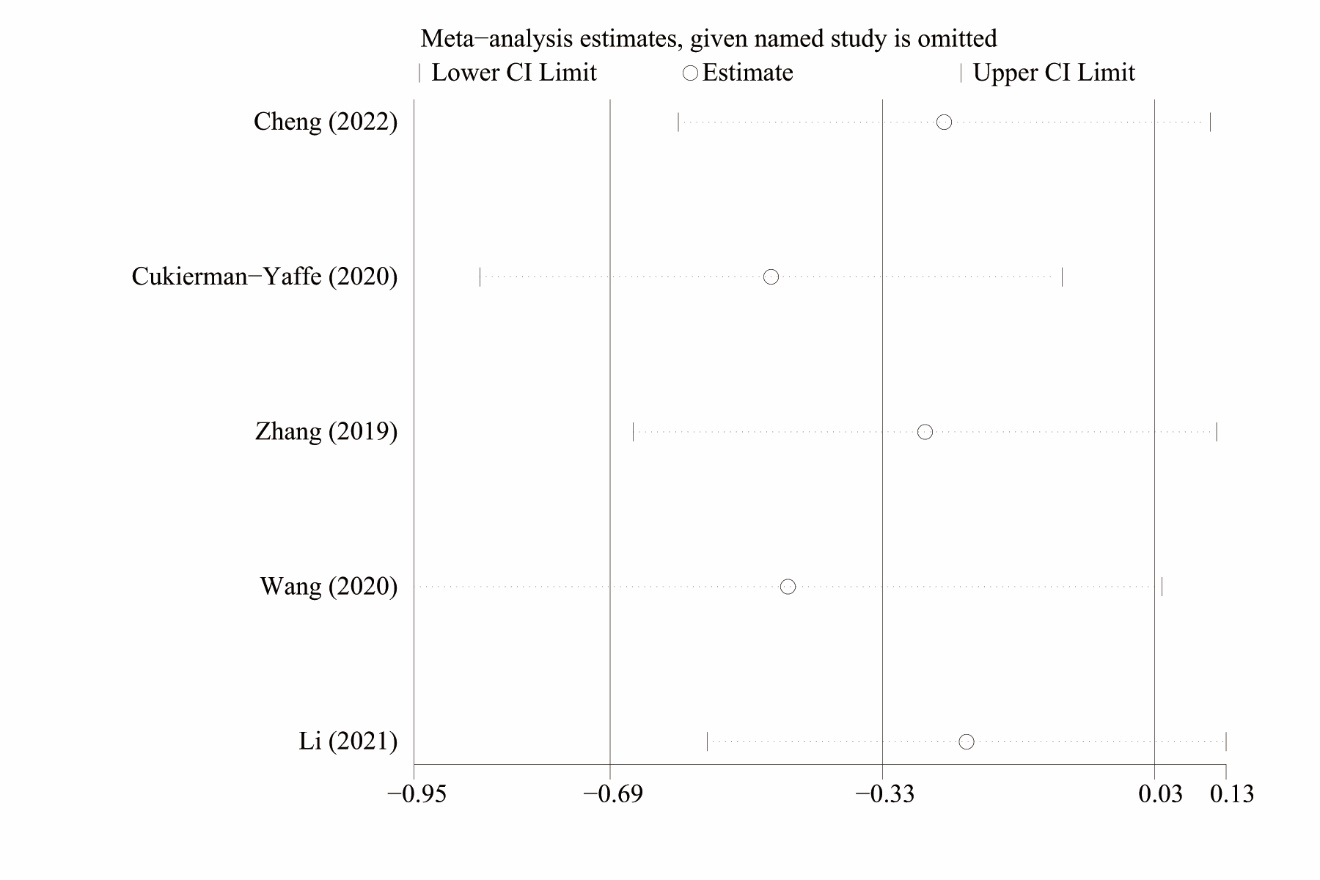


**SFigure.3 Sensitivity analyses of self-controlled studies.**


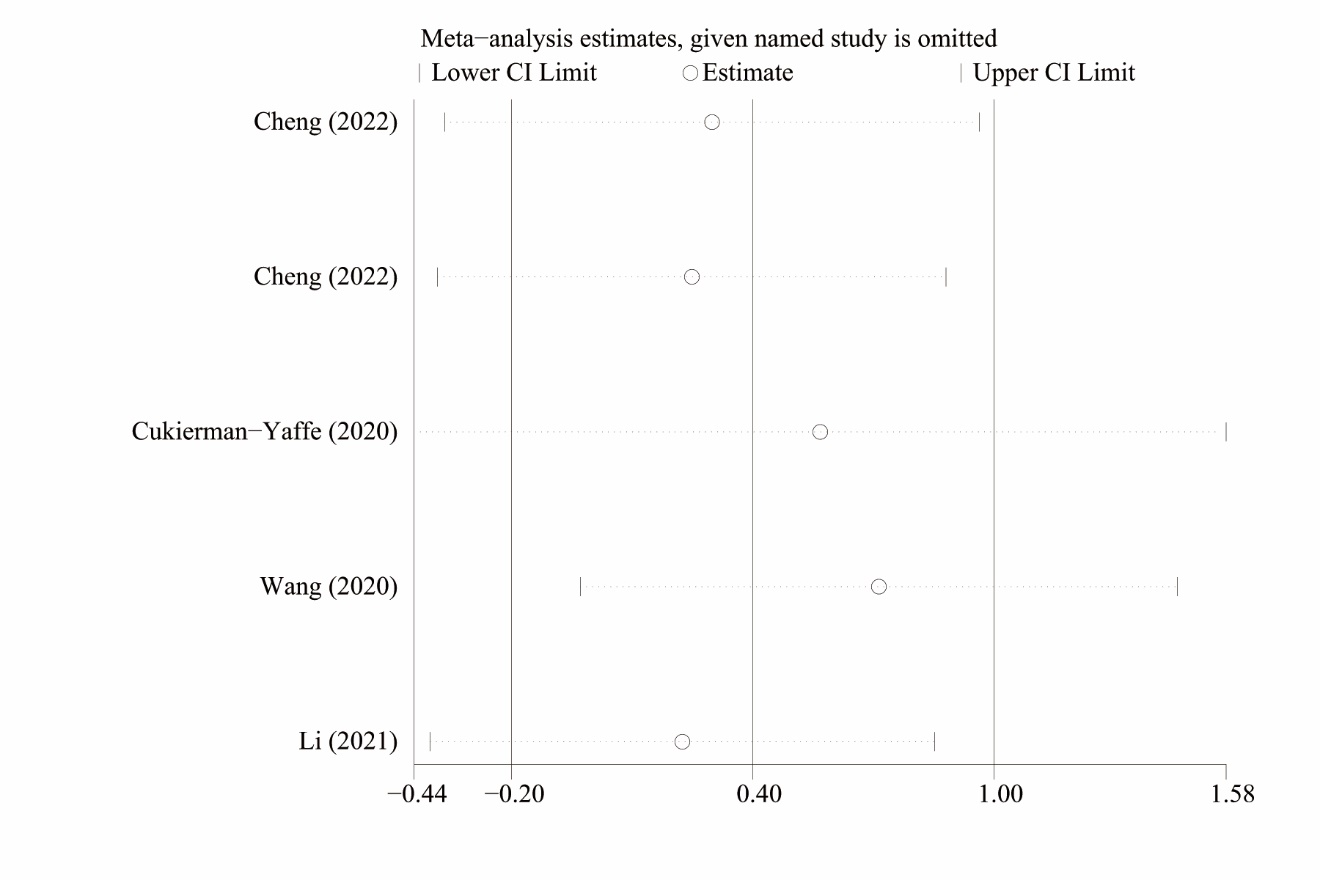


**SFigure.4 Sensitivity analyses of** **non-GLP-1 analog-treated controlled studies.**
